# Endoscopic hand suturing with clips for a large defect after endoscopic full-thickness resection of gastric gastrointestinal stromal tumor

**DOI:** 10.1055/a-2299-2189

**Published:** 2024-05-17

**Authors:** Zhenkun Wu, Yong Liu, Shibo Song, Wenyu Li, Hoiloi Ng, Shun He, Guiqi Wang

**Affiliations:** 1 Endoscopy, National Cancer Center/National Clinical Research Center for Cancer/Cancer Hospital, Chinese Academy of Medical Sciences and Peking Union Medical College, Beijing, China

Few studies have focused on endoscopic full-thickness resection (EFTR) for gastric gastrointestinal stromal tumors (g-GISTs) ≥35 mm 1 , which could be attributed to the difficulty of endoscopic resection and closure of the defect, although multiple closure techniques have been developed for post-EFTR defects 2 3 4 . Recently, endoscopic hand-suturing (EHS) has been proved safe and effective for gastrointestinal superficial defects 5 , and can be expected to be similarly efficacious for closing large defects after EFTR. Here, we describe a successful case of full-thickness closure using EHS with clips (EHS-Clips) for a large g-GIST defect.

A 72-year-old man who underwent gastroscopy was diagnosed with a submucosal tumor approximately 4.0 × 3.5 cm in size at the fundus ( Fig. 1 ). Endoscopic ultrasonography and contrast-enhanced computerized tomography suggested a g-GIST ( Fig. 2 ). After comprehensive multidisciplinary discussions and thorough communication with the patient, the lesion was removed en bloc through EFTR, leaving a large full-thickness defect ( Fig. 3 ). The defect was completely sutured via EHS, and this was followed by the application of clips for additional mucosal closure, to enhance the reliability of closure and ensure patient safety ( Fig. 4 , Fig. 5 , Video 1 ). The abdominal gas accumulation was released through abdominal puncture. The resection time and suture time were 33 minutes and 60 minutes respectively. On postoperative day 5, a follow-up endoscopy confirmed continued closure, allowing discharge of the patient. No adverse events occurred during or after the operation. Histologically, complete resection of a very low risk g-GIST had been obtained.

**Fig. 1:**
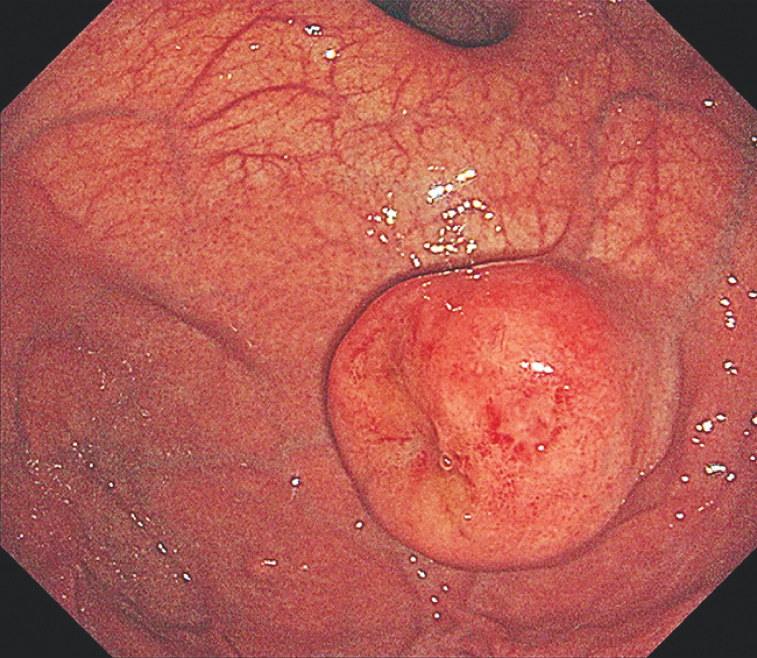
Gastroscopy in a 72-year-old man showed a gastric submucosal tumor at the fundus, approximately 4.0 × 3.5 cm in size.

**Fig. 2:**
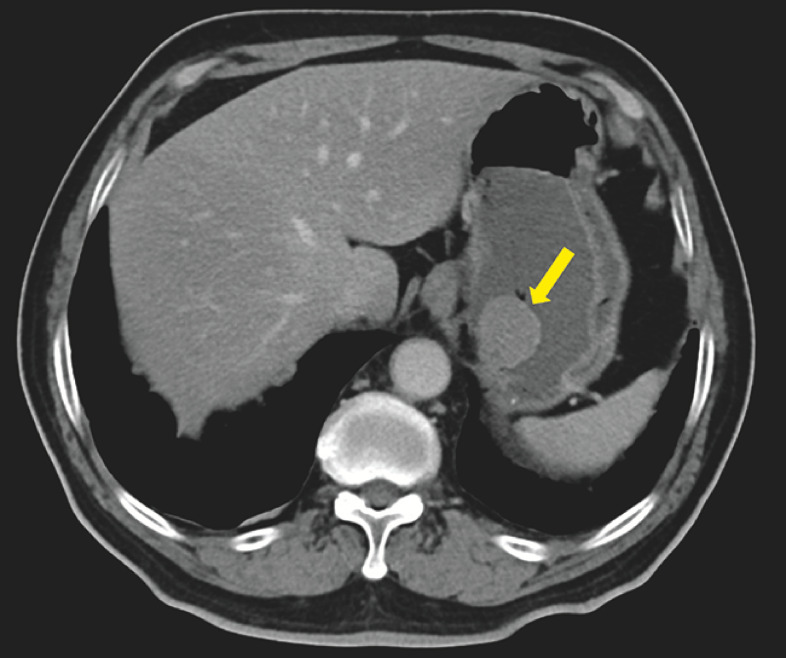
Contrast-enhanced computerized tomography suggested a gastric gastrointestinal stromal tumor (g-GIST) (yellow arrow).

**Fig. 3:**
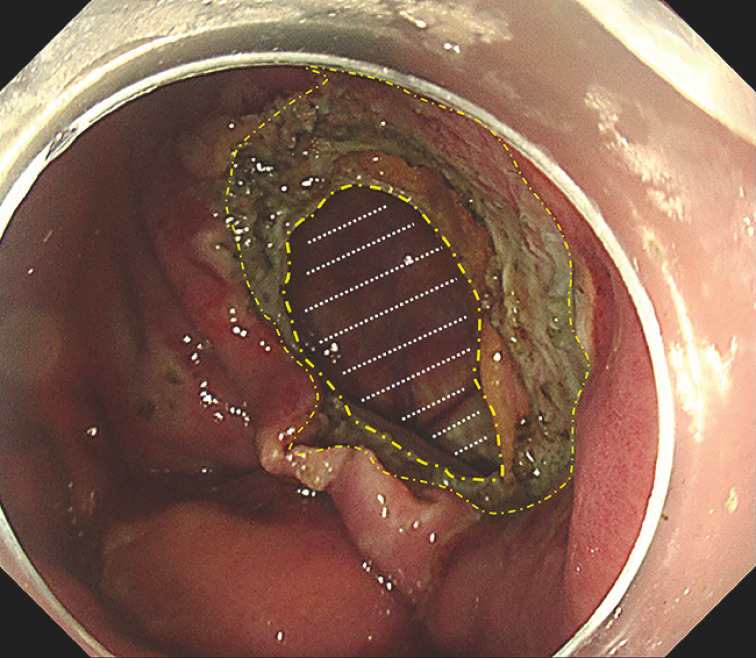
Defect after endoscopic full-thickness resection (EFTR) of a g-GIST at the fundus. The yellow dash-dotted lines outline the defect; the white dotted lines indicate the diaphragm.

**Fig. 4:**
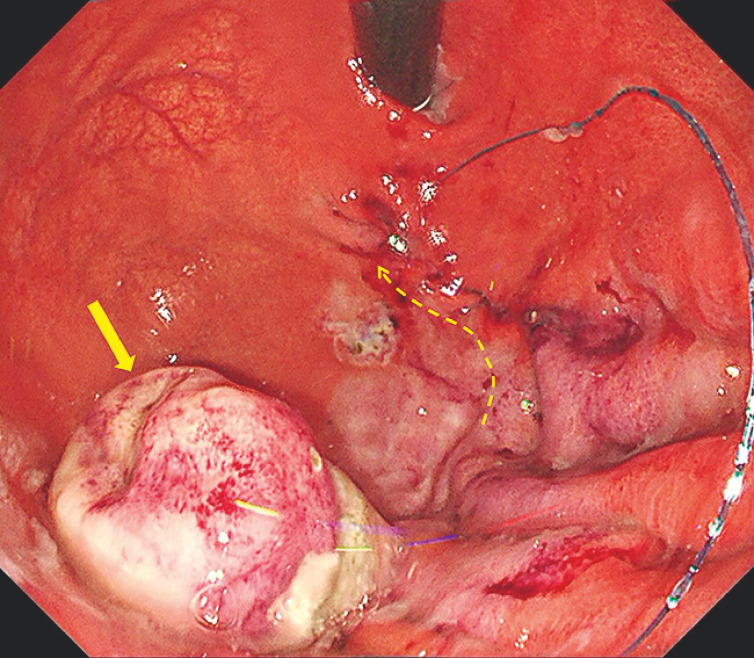
The defect after closure by endoscopic hand-suturing (EHS). Dotted line and arrow, suture direction; arrow, resected tumor.

**Fig. 5:**
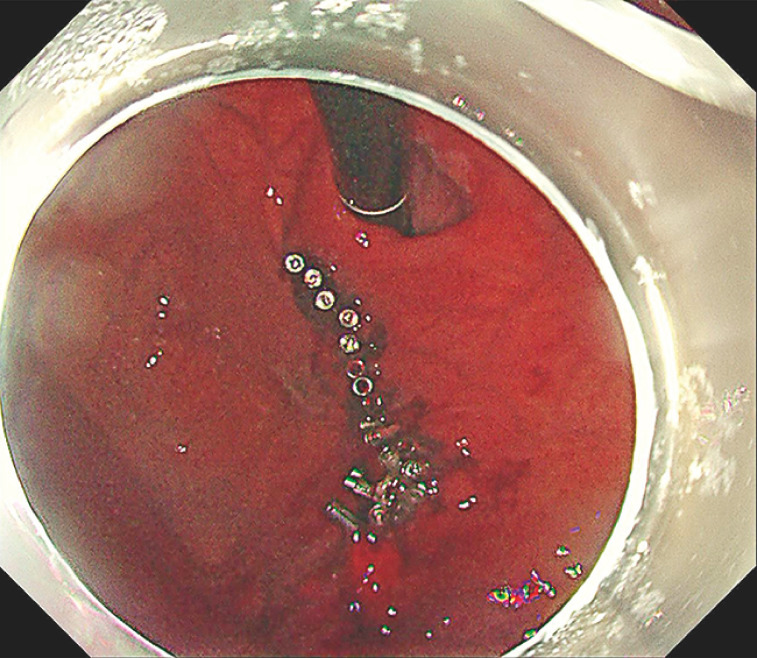
Additional clips, applied for reliable closure and patient safety.

Complete closure of a large defect after endoscopic full-thickness resection (EFTR) of a gastric gastrointestinal stromal tumor (g-GIST), using endoscopic hand-suturing followed by clipping (EHS-Clips) for additional mucosal closure, to enhance reliability of closure and ensure patient safety.Video 1

To our knowledge, this EHS-Clips case is the first such report of complete closure of a large full-thickness defect. Notably, the suturing process should ensure the protection of adjacent organs and tissues from injury. Therefore, the EHS-Clips approach can be considered as an optional closure method for full-thickness defect after EFTR in selected patients. Further accumulation of clinical experience is needed.

Endoscopy_UCTN_Code_TTT_1AO_2AZ

## Notes

Endoscopy E-Videos https://eref.thieme.de/e-videos
*E-Videos* is an open access online section of the journal *Endoscopy* , reporting on interesting cases and new techniques in gastroenterological endoscopy. All papers include a high-quality video and are published with a Creative Commons CC-BY license. Endoscopy E-Videos qualify for HINARI discounts and waivers and eligibility is automatically checked during the submission process. We grant 100% waivers to articles whose corresponding authors are based in Group A countries and 50% waivers to those who are based in Group B countries as classified by Research4Life (see: https://www.research4life.org/access/eligibility/ ). This section has its own submission website at https://mc.manuscriptcentral.com/e-videos .
